# The CD8-Derived Chemokine XCL1/Lymphotactin Is a Conformation-Dependent, Broad-Spectrum Inhibitor of HIV-1

**DOI:** 10.1371/journal.ppat.1003852

**Published:** 2013-12-26

**Authors:** Christina Guzzo, Jamie Fox, Yin Lin, Huiyi Miao, Raffaello Cimbro, Brian F. Volkman, Anthony S. Fauci, Paolo Lusso

**Affiliations:** 1 Laboratory of Immunoregulation, National Institute of Allergy and Infectious Diseases, National Institutes of Health, Bethesda, Maryland, United States of America; 2 Department of Biochemistry, Medical College of Wisconsin, Milwaukee, Wisconsin, United States of America; University of Zurich, Switzerland

## Abstract

CD8+ T cells play a key role in the *in vivo* control of HIV-1 replication via their cytolytic activity as well as their ability to secrete non-lytic soluble suppressive factors. Although the chemokines that naturally bind CCR5 (CCL3/MIP-1α, CCL4/MIP- 1β, CCL5/RANTES) are major components of the CD8-derived anti-HIV activity, evidence indicates the existence of additional, still undefined, CD8-derived HIV-suppressive factors. Here, we report the characterization of a novel anti-HIV chemokine, XCL1/lymphotactin, a member of the C-chemokine family that is produced primarily by activated CD8+ T cells and behaves as a metamorphic protein, interconverting between two structurally distinct conformations (classic and alternative). We found that XCL1 inhibits a broad spectrum of HIV-1 isolates, irrespective of their coreceptor-usage phenotype. Experiments with stabilized variants of XCL1 demonstrated that HIV-1 inhibition requires access to the alternative, all-β conformation, which interacts with proteoglycans but does not bind/activate the specific XCR1 receptor, while the classic XCL1 conformation is inactive. HIV-1 inhibition by XCL1 was shown to occur at an early stage of infection, via blockade of viral attachment and entry into host cells. Analogous to the recently described anti-HIV effect of the CXC chemokine CXCL4/PF4, XCL1-mediated inhibition is associated with direct interaction of the chemokine with the HIV-1 envelope. These results may open new perspectives for understanding the mechanisms of HIV-1 control and reveal new molecular targets for the design of effective therapeutic and preventive strategies against HIV-1.

## Author Summary

Although HIV, the causative agent of AIDS, establishes a lifelong infection that cannot be eradicated even with effective treatment, the host immune system has the ability to contain its replication for many years in which the disease remains asymptomatic. Key players in HIV control are CD8+ T cells, specialized immune cells that can not only destroy infected cells, but also secrete soluble factors that suppress the virus without killing infected cells. CD8+ T cells produce multiple HIV-suppressive factors, including certain chemokines (soluble proteins that attract immune cells), which block the virus even before it can gain access to its target cells. In the present study, we characterize a new anti-HIV chemokine, XCL1 or lymphotactin, which is primarily produced by CD8+ T cells. A unique feature of XCL1 is that, unlike other antiviral chemokines, it has a very broad spectrum of activity against different variants of HIV-1 and directly binds the virus outer coat, rather than blocking specific receptors on the target cell. Also unique is that fact that XCL1 adopts two possible conformations, and only one of them is capable of HIV inhibition. These findings may open new avenues for the design of effective drugs or vaccines against HIV.

## Introduction

The replication of HIV-1 is regulated *in vivo* by a complex network of cytokines and chemokines expressed by immune and inflammatory cells. Key players in the mechanisms of HIV-1 control are CD8+ T cells, which, in addition to their cytolytic activity, secrete soluble factors that suppress HIV-1 in a non-lytic fashion [1]–[5]. Following the initial observation of this latter phenomenon in 1986 by Walker and colleagues [4], subsequent studies demonstrated HIV-1 inhibition in co-cultures of CD8+∶CD4+ T cells separated by a semi-permeable membrane, as well as in cell-free supernatants from activated CD8+ T cells [1], [3], thus, ruling out the need for cell-to-cell contact. Moreover, the ability of CD8+ T cells to suppress HIV-1 replication was shown to correlate with the clinical stage of HIV-1 infection, suggesting a potential *in vivo* protective effect of this non-lytic CD8+ T cell activity [5]. Approximately 10 years after the initial description of soluble CD8+ T cell-derived inhibition of HIV replication, three chemokines of the CC (β) chemokine family (CCL3/MIP-1α, CCL4/MIP- 1β, CCL5/RANTES) were identified as major components of the soluble CD8+ T cell-derived anti-HIV activity [2]. These three chemokines act via a redundant mechanism of binding and downmodulating CCR5 to block entry of viruses with CCR5 coreceptor tropism. However, multiple lines of evidence indicate the existence of additional, still undefined, CD8-derived factors that can suppress HIV-1 infection. In particular, the observation that CD8^+^ T-cell culture supernatants can also inhibit CC-chemokine-resistant HIV-1 strains, such as those restricted to CXCR4 coreceptor usage [6], [7], substantiates a role for new, still uncharacterized anti-HIV factors produced by CD8^+^ T cells. Additionally, several reports have documented a suppressive effect of these factors at the transcriptional level [8]–[10], whereas CCR5-binding chemokines act at the level of viral entry/fusion.

In addition to CD8^+^ T cells, other cells of the immunohematological system can produce soluble HIV-suppressive factors, including CD4^+^ T cells, γ/δ T cells, NK cells, cells of the mononuclear phagocytic system, and platelets [11]–[13]. Recently, we identified a novel antiviral chemokine, CXCL4/PF4, which is mainly produced by megakaryocytes and platelets. CXCL4 was shown to inhibit a broad spectrum of HIV-1 isolates, irrespective of their coreceptor usage and genetic subtype; it acts at the level of viral entry via a novel mechanism mediated by direct interaction with the viral envelope [14].

In this study, we report the characterization of a novel anti-HIV-1 C-chemokine, XCL1, which exhibits a broad spectrum of activity against different biological variants of HIV-1. We present evidence that this chemokine blocks infection at an early step of the viral life cycle, namely, viral attachment and entry into host cells. Similar to our previous work with CXCL4/PF4, we found that XCL1 acts through an unconventional mechanism mediated by direct interaction with the HIV-1 envelope. Moreover, we investigated the correlation between the unique metamorphic properties of XCL1 [15] and its antiviral activity, showing that the alternatively-folded (all β-sheet) structure of XCL1 is the specific conformation responsible for HIV-1 blockade. These results offer insights into pathogenesis and provide new molecular targets for HIV-1 therapy and vaccine development.

## Results

### Primary human CD8^+^ T cells secrete XCL1/lymphotactin

**Figure 1:**
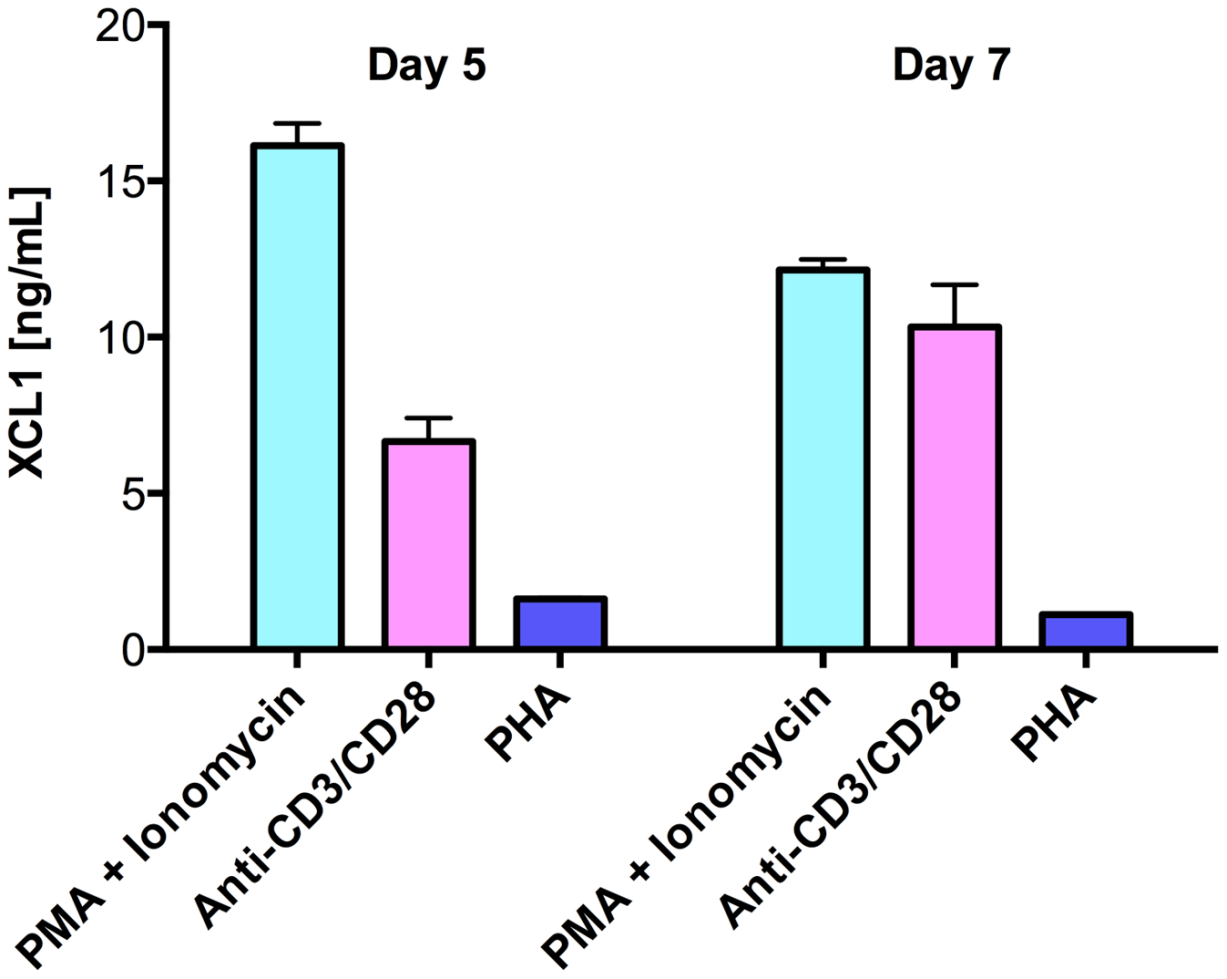
Primary human CD8+ T cells secrete XCL1. CD8+ T cells were activated with three different stimuli for 3 days: PMA plus ionomycin, anti-CD3/CD28 loaded beads, or PHA. Cells were washed and culture supernatants were harvested after 5 and 7 days to quantify XCL1 secretion.

As multiple lines of evidence indicated the existence of still uncharacterized HIV-suppressive factors produced by CD8^+^ T cells [16], we used a wide-platform cytokine array (RayBio), which evaluates in a semi-quantitative fashion 507 soluble factors, to screen culture supernatants from activated primary human CD8^+^ T cells. Among the top 25% most expressed proteins, we identified the C-chemokine XCL1/lymphotactin, in addition to other previously reported anti-HIV chemokines (Guzzo et al., in preparation). We focused our attention on XCL1 because it is produced preferentially by CD8^+^ T cells [17], [18]. Production of XCL1 by primary human CD8^+^ T cells was confirmed by ELISA in culture supernatants from CD8^+^ T cells activated *ex vivo* with either PHA, PMA plus ionomycin, or anti-CD3/CD28 antibodies (Figure 1). PMA plus ionomycin was the most potent stimulation for XCL1 production, followed by anti-CD3/CD28 antibodies, while PHA elicited the secretion of markedly lower levels.

### XCL1 inhibits a broad range of HIV-1 isolates irrespective of coreceptor-usage phenotype

**Figure 2:**
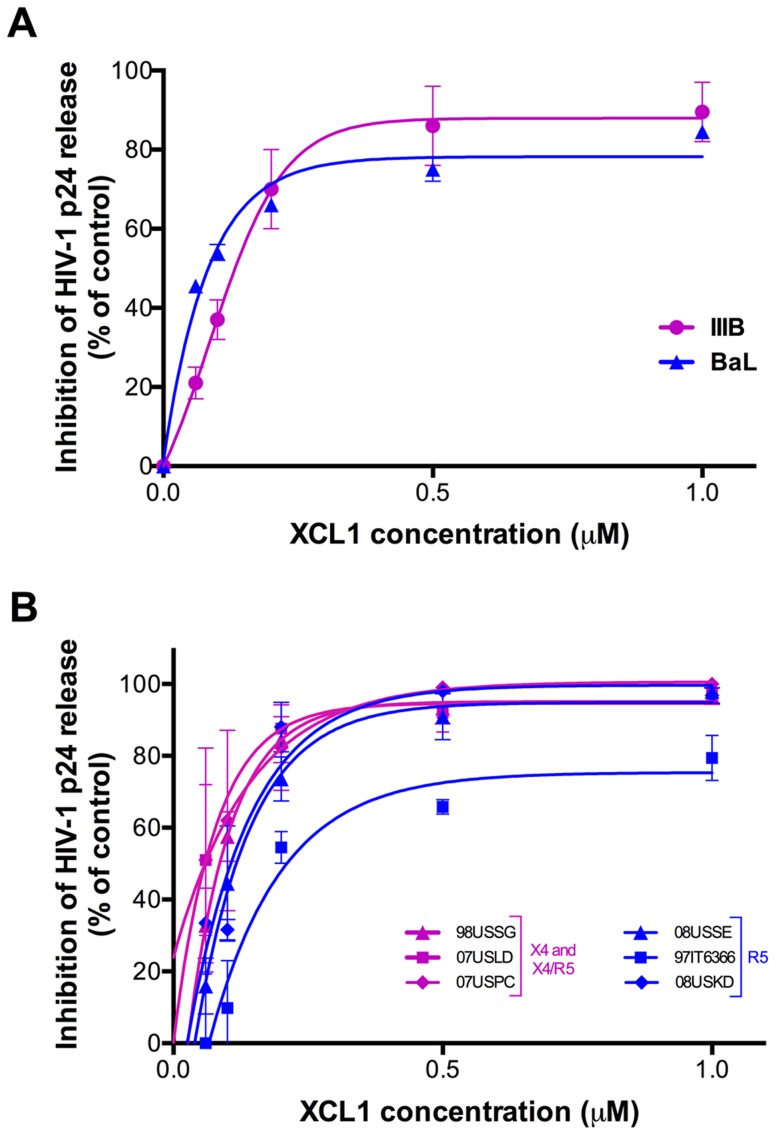
XCL1 inhibits HIV-1 infection irrespective of coreceptor usage. (A) Dose-dependent inhibition of prototypic X4 (magenta) and R5 (blue) HIV-1 strains (IIIB and BaL) by recombinant XCL1 (Peprotech) in PBMC. Virus replication was assessed by measuring the amount of p24 Gag antigen in culture supernatants via AlphaLISA immunoassay. Data were normalized to the amount of viral replication observed in control cultures (not treated with XCL1). Data represent the mean values (±SD) of replicate wells, representative of at least 5 independent experiments performed on separate PBMC donors. (B) Similar infection protocols were performed on a larger panel of primary HIV-1 isolates with different coreceptor specificity. Inhibition curves in magenta represent CXCR4- and CXCR4/CCR5-tropic isolates, while those in blue represent CCR5-tropic viruses.

To assess the ability of XCL1 to inhibit HIV-1 infection, acute infection assays were initially performed with recombinant human XCL1 (Peprotech) in primary human PBMC infected with laboratory-adapted viral strains. XCL1 potently inhibited HIV-1 infection irrespective of coreceptor specificity, as it was equally effective on strains specific for CXCR4 (X4; IIIB) and CCR5 (R5; BaL) (Figure 2A). To further examine the breadth of XCL1-mediated inhibition, we evaluated the ability of recombinant XCL1 to inhibit infection of primary PBMC with a panel of primary HIV-1 isolates with different coreceptor-usage specificity. As seen with the laboratory-adapted HIV-1 strains, XCL1 was equally effective on CCR5-specific and CXCR4-using primary isolates (Figure 2B). Of note, XCL1 did not reach 90% inhibition on two R5 isolates even at the highest dose used (1 µM). Treatment with XCL1 did not reduce cell viability, indicating that the HIV-inhibitory effect of XCL1 was not due to toxic or anti-metabolic effects on the cells (data not shown). The anti-HIV-1 activity of XCL1 was also confirmed in the engineered cell line TZM-bl [19]–[21], a HeLa cell derivative expressing CD4, CXCR4, and CCR5 (data not shown).

### The alternatively-folded, all-β conformation of XCL1 is active against HIV-1

**Figure 3:**
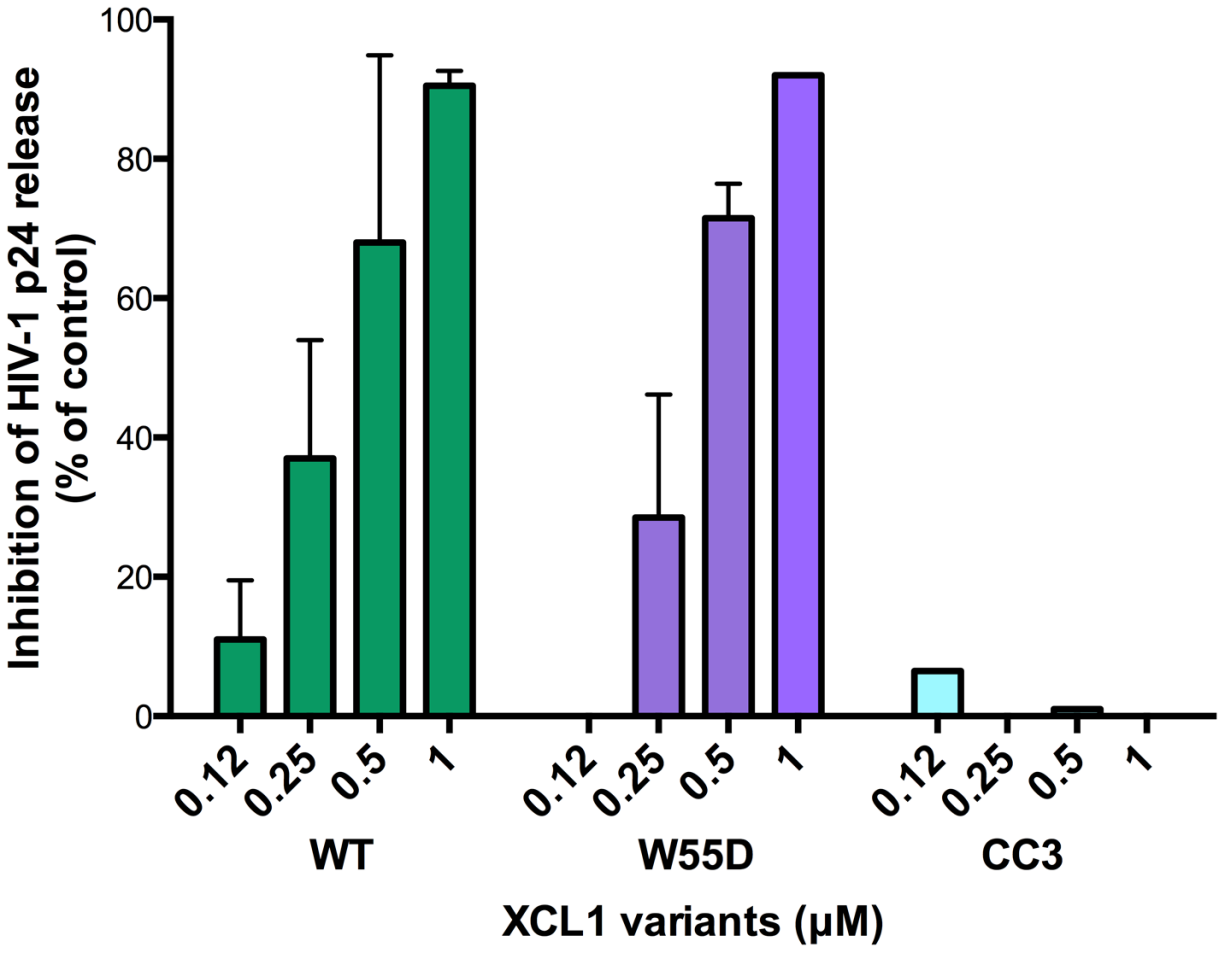
The inhibitory structure of XCL1 against HIV-1 is the all-β, alternatively-folded conformation. Dose-dependent inhibition of an XCL1-sensitive primary HIV-1 isolate (92HT599; X4/R5) by recombinant wild type (WT) XCL1 (green bars), and two stabilized structural variants: W55D, a variant preferentially adopting the all-β/alternatively-folded structure (purple bars), and CC3, a variant locked in the classical chemokine-folded structure (light blue bars). Virus replication was assessed by measuring the amount of p24 Gag antigen in PBMC supernatants via AlphaLISA immunoassay. Data were normalized to the amount of viral replication observed in control cultures (not treated with XCL1). Data represent the mean values (±SD) of replicate wells, representative of at least 3 independent experiments performed on separate PBMC donors.

XCL1 is a metamorphic chemokine that interconverts in solution between two distinctly folded structures, namely the canonical chemokine fold (three-stranded anti-parallel β-sheet and C-terminal helix), which has been reported to bind and activate the specific XCL1 receptor (XCR1), and an alternative, four-stranded sheet that forms a dimeric β sandwich, which was reported to bind glycosaminoglycans (GAGs), but not XCR1 [15]. Thus, we tested two stabilized recombinant XCL1 variants produced in *E. coli*: CC3, a variant locked in the chemokine-folded structure [22], and W55D, a variant that preferentially adopts the alternatively-folded dimeric structure [15]. As a control, we also tested a full-length, wild type (WT) XCL1, which retains the ability to interconvert between protein folds. A dual-tropic primary HIV-1 isolate sensitive to XCL1-mediated inhibition, 92HT599, was used for acute infection assays to evaluate inhibition with XCL1 variants. As shown in Figure 3, we observed striking differences in the inhibition exhibited by the locked XCL1 variants, CC3 and W55D. Indeed, the W55D variant inhibited HIV-1 with similar potency, as did the WT chemokine, while the CC3 variant showed no appreciable inhibition at the doses used. We observed similar results in infection assays performed with both CXCR4-tropic (IIIB) and CCR5-tropic (BaL) HIV-1 isolates, with the all-β, alternatively-folded conformation (W55D) conferring inhibition, and the chemokine-folded conformation (CC3) showing minimal, if any, activity (Figure S1). These results suggested that the antiviral activity of XCL1 is dependent on the protein conformation and appears to be unrelated to activation of the XCR1 receptor, which is specifically bound and activated by the CC3 variant, but not by the W55D variant. In line with this observation, we were unable to detect XCR1 expression by flow cytometry in the HIV-1 target cells used in our study (data not shown).

### XCL1 blocks HIV-1 attachment and entry

**Figure 4:**
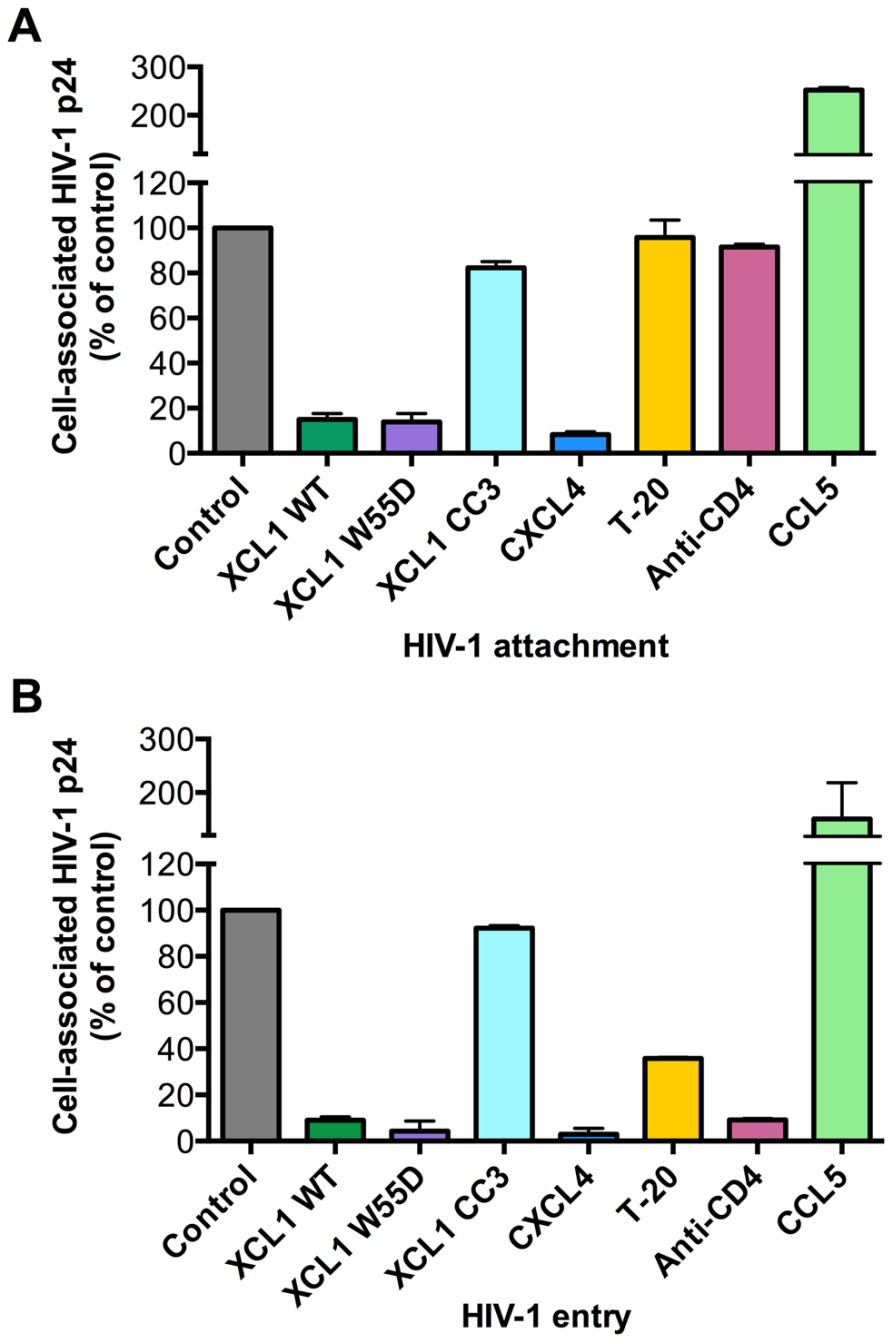
XCL1 blocks HIV-1 attachment and entry into target cells. Both attachment and entry assays were performed on TZM-bl cells incubated with the XCL1-sensitive dual tropic HIV-1 strain, 92HT599. (A) Virus attachment was measured as the total amount of cell-associated p24 Gag protein after 4 h incubation with virus at 37°C and subtraction of background levels. Background attachment/entry was quantified as the cell-associated p24 Gag after incubation of cells with virus at 4°C and subsequent trypsin treatment. (B) Virus entry assay was performed in a similar manner except cells were incubated with virus for 2 additional hours (6 h total) and trypsinized to remove extracellular-associated virus. Cells were lysed and virus entry was determined as the total amount of trypsin-resistant, intracellular p24 protein. Recombinant cytokines were used at 15 µg/mL, the T-20 fusion inhibitor at 50 µg/mL, and anti-CD4 antibody at 20 µg/mL. The data represent mean values (±SD) of replicate wells from at least 3 independent experiments.

Since we established that the anti-HIV activity of XCL1 is associated with XCL1 adopting the all-β (alternatively-folded) conformation, which does not bind and activate the XCR1 receptor but is capable of interacting with GAGs [23], we focused on the extracellular events in the HIV-1 replication cycle and assessed the ability of XCL1 to interfere with viral attachment and entry. To achieve a high level of consistency of these assays, experiments were performed using TZM-bl cells infected with the primary dual-tropic isolate, 92HT599, which is highly sensitive to XCL1-mediated inhibition. As seen in Figure 4A and B, the WT chemokine and the W55D variant effectively blocked viral attachment and entry, while the CC3 variant had no appreciable inhibitory effect, reflecting the pattern of inhibition seen in the infection assays (Figures 2 and 3). In parallel, we examined the inhibitory effects of two other anti-HIV chemokines, namely CXCL4/PF4 and CCL5/RANTES. In accordance with our previous work, CXCL4 effectively inhibited viral attachment and entry [14], whilst CCL5 had enhancing effects, as previously observed with other CXCR4-tropic HIV-1 strains [24]. Additional controls included T-20, a well-characterized fusion inhibitor [25], which showed no effect on viral attachment, but a significant reduction in viral entry, and an anti-CD4 monoclonal antibody (mAb), which showed only a slight reduction in attachment but a very marked inhibition of viral entry (Figure 4A,B).

### XCL1 does not modulate the expression of the major HIV-1 receptors

Since we documented an inhibitory effect of XCL1 at the level of HIV-1 attachment and entry, we examined the ability of XCL1 to downmodulate the main HIV-1 cellular receptors, namely, CD4, CXCR4 and CCR5. Flow cytometry did not reveal any change in surface expression of these receptors after XCL1 treatment for 24 h (data not shown). Furthermore, we also investigated interactions between XCL1 and CD4 via binding assays with anti-CD4 antibodies targeting different domains of CD4 (D1, D2 and D3–4); we did not observe any modification in CD4 staining, suggesting that XCL1 does not interact with cell surface-expressed CD4 (data not shown).

### XCL1 directly interacts with the HIV-1 envelope glycoprotein, gp120

**Figure 5:**
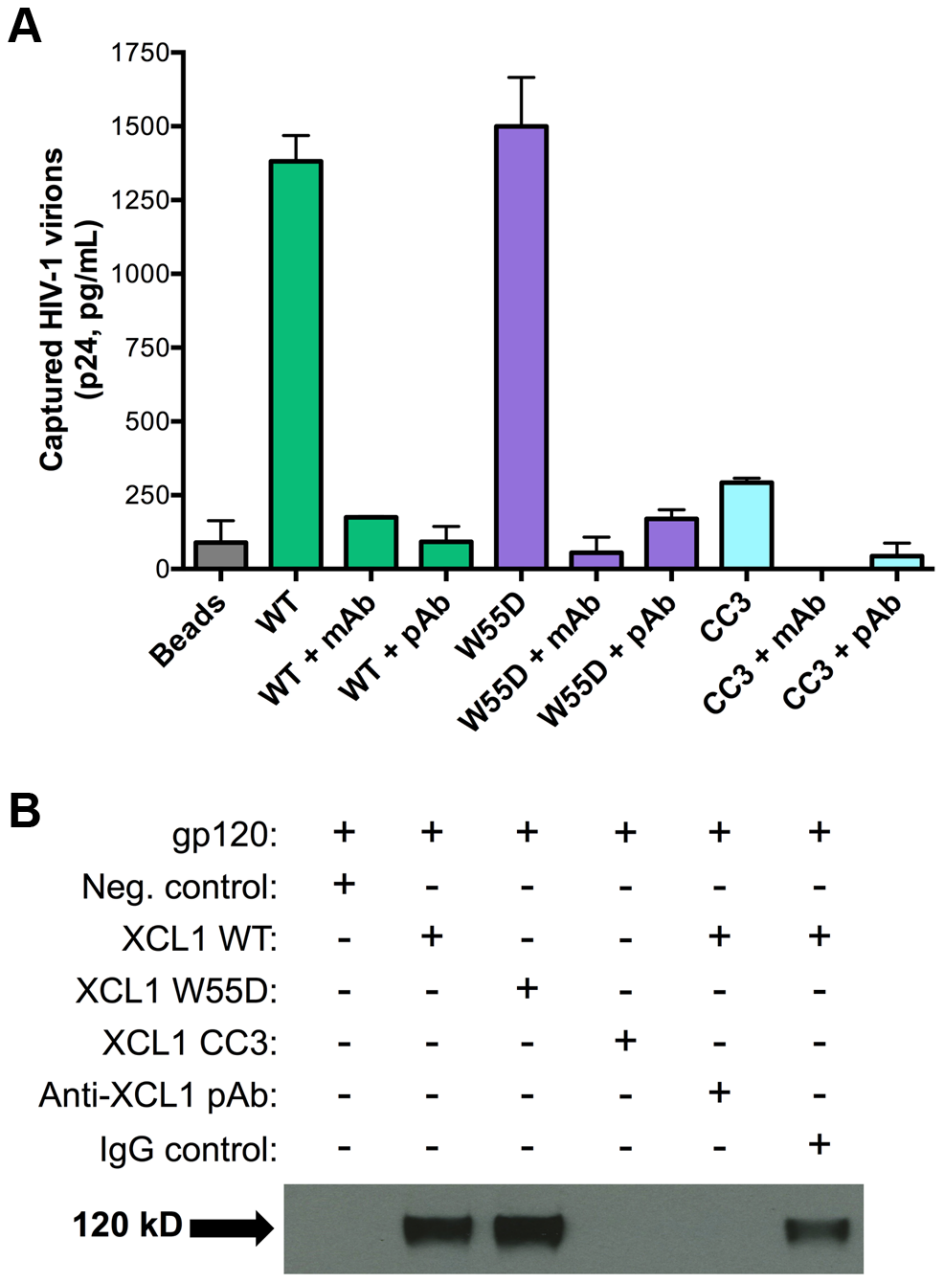
XCL1 captures native HIV-1 virions and interacts directly with the external viral envelope glycoprotein, gp120. (A) The virion capture assay was performed using immunomagnetic beads armed with 3 different XCL1 variants (WT in green bars, W55D in purple bars, and CC3 in light blue bars) as molecular baits to capture whole HIV-1 virions (strain IIIB; X4). The specificity of the interaction was demonstrated upon pre-incubation of XCL1-armed beads (prior to virus addition) with 20 µg/mL of monoclonal (mAb) and polyclonal (pAb) anti-XCL1 antibodies. (B) Co-immunoprecipitation of the HIV-1 envelope glycoprotein gp120 observed with XCL1 WT and W55D, but not CC3. From left to right: lane 1 showing a negative-control (unconjugated XCL1 WT plus gp120); lane 2 with biotinylated XCL1 WT and gp120 co-precipitated; lane 3 with biotinylated XCL1 W55D and gp120 co-precipitated; lane 4 with no co-precipitation of biotinylated XCL1 CC3 and gp120; lane 5 with complete inhibition of XCL1 WT-gp120 co-precipitation in the presence of anti-XCL1 pAb; and lane 6 with IgG control showing minimal inhibition on XCL1-gp120 co-precipitation.

In view of the data described above, we investigated the possibility that XCL1 may interact directly with HIV-1 virions, as we previously demonstrated for CXCL4/PF4 [14]. To investigate this hypothesis, we performed a virion capture assay by which immunomagnetic beads were armed with different XCL1 variants (WT, W55D, or CC3) as molecular “baits” to capture whole HIV-1 virions, as previously described [14]. Figure 5A shows that both WT and W55D efficiently captured HIV-1 virions. The specificity of this interaction was validated upon observation of reduced capture when XCL1-armed beads were pre-incubated with anti-XCL1 mAb or polyclonal antibody (pAb) prior to exposure to the virus. In agreement with our infection and attachment/entry assays, we did not observe any appreciable virion capture when the beads were armed with the CC3 XCL1 variant. Our data demonstrate that XCL1 can directly interact with HIV-1 virions, and that the all-β (alternatively-folded) XCL1 conformation (W55D) mediates this interaction, while the classic chemokine-folded conformation (CC3) does not. To support the relevance of these data to the antiviral activity of XCL1, we found that the same anti-XCL1 pAb that was used to block HIV-1 capture reversed the antiviral activity of XCL1 in PBMC infection experiments (Figure S2).

To demonstrate that XCL1 can interact directly with the external viral envelope glycoprotein, gp120, we performed co-immunoprecipitation studies with biotin-conjugated XCL1 WT and variants. As seen in Figure 5B, XCL1 WT and W55D were able to specifically co-immunoprecipitate gp120, while the CC3 variant did not. Importantly, the same anti-XCL1 pAb that prevented virion capture abrogated gp120 co-immunoprecipitation (Figure 5B). Taken together, these data support a mechanism of HIV-1 inhibition whereby XCL1 interacts with viral particles via direct binding to the external viral envelope glycoprotein, gp120. Furthermore, these data confirm the dependency of the anti-HIV-1 activity of XCL1 on the all-β (alternatively-folded) conformation.

### VSV-G pseudotyped virus is insensitive to XCL1-mediated inhibition and virion capture

**Figure 6:**
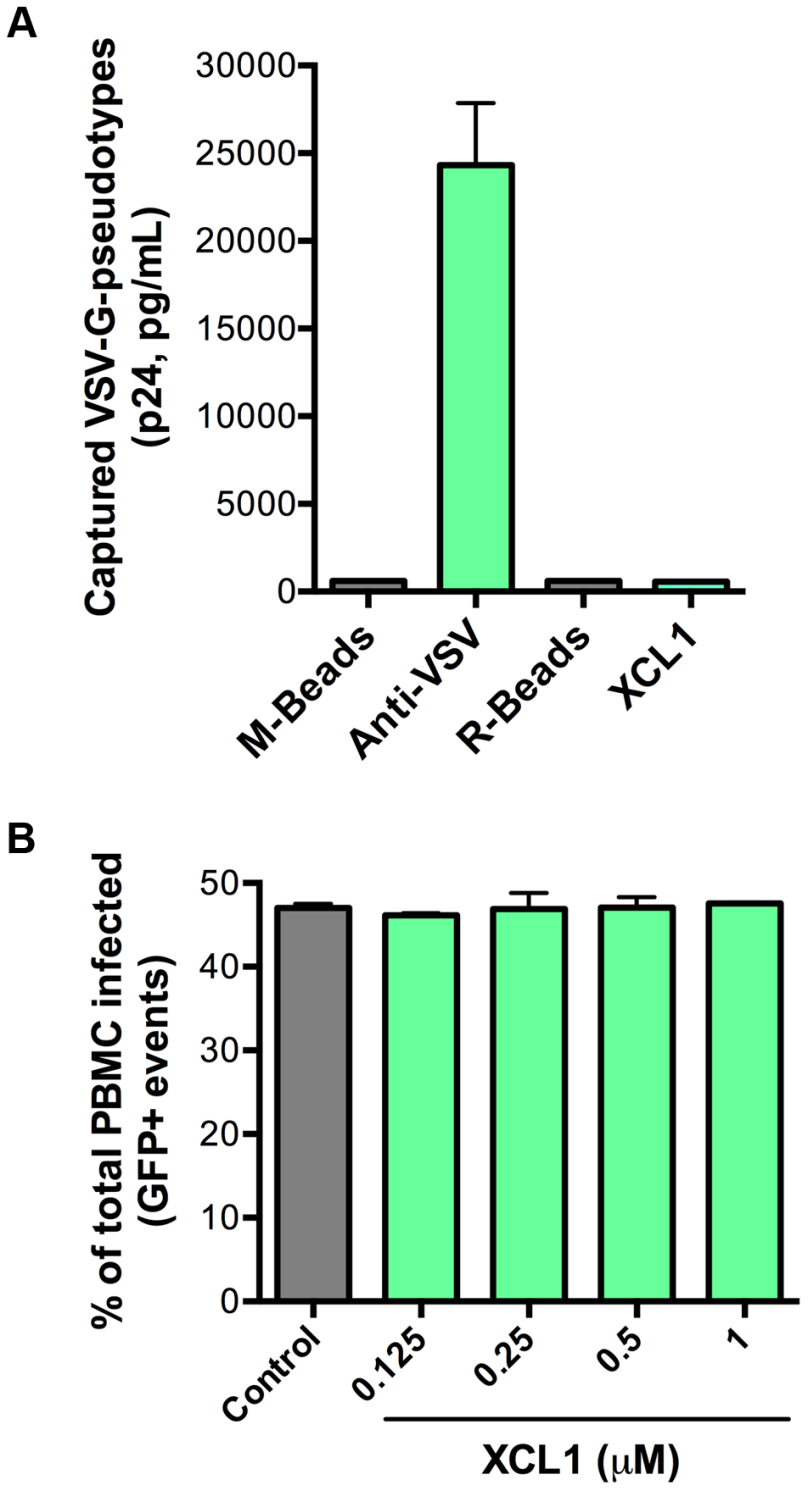
XCL1 does not interact with or inhibit VSV-G-pseudotyped virus. (A) Virion capture was performed using anti-mouse immunomagnetic beads (M-Beads) armed with anti-VSV-G mAb (Anti-VSV) or anti-rabbit immunomagnetic beads (R-Beads) armed with XCL1 WT (XCL1). Equal amounts of VSV-G-pseudotyped virus were added to all bead reactions. (B) Infection of PBMC with GFP-expressing VSV-G-pseudotyped virus was not affected by a dose-response treatment of XCL1 WT. Virus infection was quantified by the number of infected cells (GFP-positive) counted from the total PBMCs harvested from each well.

As an additional test for specificity of the interaction between XCL1 and the HIV-1 envelope glycoprotein (gp120), we performed both virion-capture and infection assays using VSV-G pseudotyped virions, which contain an HIV-1 core surrounded by the VSV envelope. Figure 6A shows that XCL1 was unable to capture VSV-G pseudotyped virions, indicating the HIV-1 capture observed in Figure 5A required the presence of the HIV-1 envelope. To verify the relevance of these observations to the antiviral activity of XCL1, we performed acute infection assays with VSV-G pseudotyped virions in primary PBMC. We did not observe any inhibitory effect of XCL1, as evidenced by measuring both the absolute numbers of infected cells (Figure 6B), and the levels of reporter gene (GFP) expression within the gated population of infected cells (Figure S3). Additionally, we observed no inhibition of VSV-G pseudotyped virus attachment or entry in TZM-bl cells (data not shown). Altogether, these results further validate that the mechanism of XCL1 inhibition is via direct interaction with the HIV-1 envelope.

### XCL1 does not require cell-surface glycosaminoglycans to inhibit HIV-1 infection

**Figure 7:**
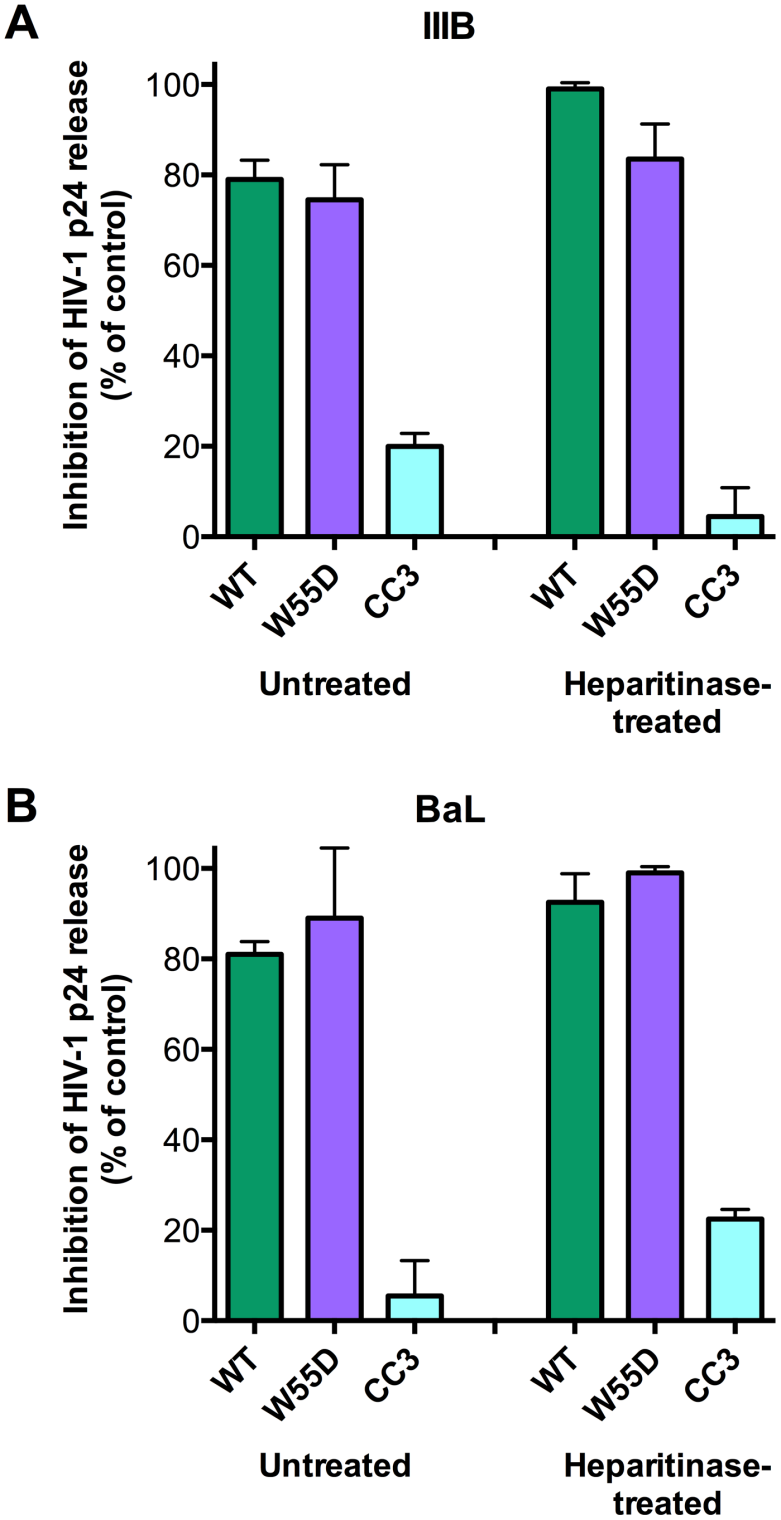
XCL1 antiviral activity is equally effective before and after digestion of glycosaminoglycans on PBMC. PBMC were incubated in the presence (‘Heparitinase-treated’) or absence (‘Untreated’) of heparitinase to digest cell surface glycosaminoglycan expression, followed by infection with HIV-1 IIIB (A) or BaL (B) in the presence of XCL1 WT (green bars), W55D (purple bars) or CC3 (light blue bars) at 1 µM. Virus replication was assessed by measuring the amount of p24 Gag antigen in PBMC supernatants via AlphaLISA immunoassay. Data were normalized to the amount of viral replication observed in control cultures (not treated with XCL1). Results represent the mean values (±SD) of replicate wells.

Since we demonstrated that the antiviral activity of XCL1 depends on the all-β conformation (W55D), previously shown to bind GAGs with high affinity [15], we examined the inhibitory activity of XCL1 in PBMC infected with X4- or R5-tropic HIV-1 following digestion of cell-surface GAGs with heparitinase. As seen in Figure 7, we observed that both WT and W55D XCL1 were equally effective at blocking HIV-1 infection in heparitinase-treated and -untreated cells, while in contrast the CC3 variant remained inactive in both conditions. The efficiency of GAG removal was evaluated by ELISA using two different anti-GAG mAbs (Figure S4). These data provide further evidence for an antiviral mechanism mediated by direct interaction of XCL1 with the viral envelope, irrespective of it's binding to GAGs and/or other structures expressed on the target cell surface.

## Discussion

In this study, we report the characterization of the CD8+ T cell-derived C-chemokine, XCL1, as a novel, broad-spectrum inhibitor of HIV-1 infection. XCL1 is primarily produced by activated CD8+ T cells and NK cells [17], and recruits T lymphocytes and dendritic cells via binding to and activation of a specific cellular receptor, XCR1 [26], [27]. A possible link between XCL1 and HIV-1 was previously suggested in two wide-screening studies of chemokines and chemokine receptors: the first reported low-level inhibition of HIV-1 replication by XCL1 [28], while the second identified a small subset of HIV-1 isolates that could use the XCL1 receptor, XCR1, as a coreceptor in cells transfected *in vitro* [29]. However, these data were not subsequently validated in further studies, nor were the potential underlying mechanisms investigated. Our work provides a thorough characterization of the anti-HIV-1 activity of XCL1, showing no apparent relationship between the antiviral action of XCL1 and the putative function of XCR1 as a coreceptor, although these findings do not exclude that XCR1 may serve as a minor HIV-1 coreceptor in specific cells or anatomical sites.

XCL1 is a unique metamorphic chemokine that can interconvert between two different conformational folds: the conserved chemokine fold (monomer), which was shown to bind to and activate XCR1, and an alternatively-folded (all β-sheet) dimeric conformation which does not activate XCR1, but instead binds to glycosaminoglycans (GAGs) with high affinity [15], [23]. Using XCL1 variants designed to predominantly fold into one of the two conformations, we found that only the alternatively-folded (all β-sheet) molecule elicited anti-HIV activity, while the chemokine locked in the classical, XCR1-interacting fold was inactive. In line with this observation, we were unable to detect XCR1 expression by flow cytometry in the HIV-1 target cells used in our study; in fact, the ability of CD4+ T cells to express XCR1 is controversial [26], [30]. At this stage, it is uncertain whether and to what extent the inherent tendency of alternatively-folded XCL1 to dimerize plays any role in HIV-1 inhibition, as a monomeric form of the alternatively-folded XCL1 is not available for testing. Regardless, since the alternatively-folded molecule binds cell-surface GAGs and not XCR1, our results led us to investigate the early events in the viral infectious cycle that take place at the target cell surface. Indeed, we documented XCL1-mediated blockade of HIV-1 at an early stage of infection, namely, viral attachment and entry. Moreover, we provide multiple lines of evidence that XCL1 inhibits HIV-1 through an unconventional mechanism mediated by direct interaction with the viral envelope, similar to that previously reported for the α-chemokine CXCL4 [14]. We showed that XCL1 efficiently captures infectious HIV-1 virions and binds to the external viral envelope glycoprotein, gp120, and that both of these interactions depend on the alternatively-folded XCL1 structure. Furthermore, the same polyclonal antibody that antagonized virion capture and gp120 binding by XCL1 also neutralized the antiviral activity of XCL1. In line with the proposed antiviral mechanism, we demonstrated that binding to cell-surface GAGs was not required for the antiviral activity of XCL1, despite the dependence on the alternative, GAG-binding conformation for HIV-1 inhibition. These findings indicate that gp120 is another selective target of the alternative XCL1 conformation in addition to GAGs.

The fact that the biologically active conformation of XCL1 against HIV-1 is the high-affinity GAG-binding structure raises several mechanistic considerations. Foremost, the amount, complexity and variability of the glycan shield that decorates the surface of gp120 most likely influences the ability of XCL1 to block HIV-1 infection, since nearly half the molecular mass of gp120 is comprised of N-linked and O-linked glycans, and changes to these carbohydrate moieties result in altered neutralization sensitivity [31]–[36]. Indeed, it is possible that XCL1 interacts with a negatively-charged domain on the surface of gp120 [37]. Future structure-function studies with mutagenized XCL1 will help delineate key domains of the chemokine that are responsible for HIV-1 inhibition.

Whether and to what extent endogenous XCL1 contributes to the mechanisms of virus control during the course of HIV-1 infection is presently unknown. Although we found that XCL1 is a broad-spectrum HIV-1 inhibitor, we observed some variability in sensitivity among HIV-1 isolates. Different degrees of sensitivity have been documented for a wide range of antiviral biomolecules, including neutralizing antibodies [38]–[42], in line with the remarkable variability of the HIV-1 envelope, which we identified as the primary target for XCL1 antiviral activity. Another unresolved question is the discrepancy (∼2-log) between the XCL1 concentrations required for HIV-1 blockade and the levels released by activated CD8+ T cells cultured *in vitro*. However, it is important to emphasize that our data were obtained with *E. coli*-produced recombinant XCL1, leading to a significant underestimation of the potency of this chemokine. In fact, the C-terminus of XCL1 is a 22-amino acid mucin-like domain containing a cluster of O-glycosylated serine and threonine residues, and previous work has demonstrated that mammalian cell-produced, fully glycosylated XCL1 exhibits approximately 2-log higher biological activity compared with non-glycosylated XCL1 produced in prokaryotic cells [43]. Currently, there are no commercial sources of mammalian cell-produced XCL1, and efforts are underway in our laboratory to produce glycosylated XCL1. In addition, it is conceivable that *in vivo*-activated CD8+ T cells may release larger amounts of XCL1 into the local microenvironment, particularly within secondary lymphoid tissues. We are currently investigating if CD8+ T cells derived from asymptomatic HIV-infected subjects produce higher concentrations of XCL1 than CD8+ T cells from uninfected subjects, in line with their reported higher production of crude antiviral factor activity [5].

The identification of the first HIV-suppressive chemokines (CCL5/RANTES, CCL3/MIP-1α and CCL4/MIP-1β) has led not only to novel insights into endogenous host defenses against HIV-1, but also to the definition of new molecular targets for antiviral drugs [44]–[47] and genetic markers of innate HIV-1 resistance [48], [49]. In a similar manner, this study could be a first step toward determining the potential physiological role of XCL1 in HIV-1 infection. Analysis of XCL1 expression in subjects that are naturally protected from HIV infection (exposed-uninfected) or from disease progression (long-term nonprogressors) may offer new insights on mechanisms of natural resistance to HIV. Furthermore, a precise identification of the XCL1-interactive surface on the viral envelope may lead to the development of novel HIV-1 entry inhibitors, as well as new molecular targets for vaccine design.

## Materials and Methods

### Cell culture and reagents

Recombinant human XCL1/lymphotactin was obtained from Peprotech (Rocky Hills, NJ); recombinant XCL1 WT and variants (CC3 and W55D) were cloned and produced by two of the authors (JF, BFV) at the Medical College of Wisconsin, Milwaukee, WI, as previously described [15]; and recombinant RANTES/CCL5 and CXCL4/PF4 were purchased from R&D Systems (Minneapolis, MN). Molar values were calculated based on the molecular weight of the monomeric chemokines. PBMC from healthy donors were activated with phytohemagglutinin (PHA; Sigma, St. Louis, MO) and recombinant human IL-2 (Roche Applied Science, Mannheim, Germany) in complete RPMI medium (Invitrogen, Carlsbad, CA), containing 10% fetal bovine serum (FBS, Hyclone, Thermo Scientific, Waltham, MA), glutamine at 2 mM, streptomycin at 50 µg/mL, and penicillin at 100 U/mL for 72 hr prior to HIV-1 infection. Cell surface glycosaminoglycan (heparin sulfate) digestion was performed by incubating PBMC (1×10^6^ cells/mL) with heparitinase (Heparinase III, Sigma) at 2 U/mL for 2 hr at 37°C in recommended buffer (20 mM Tris-HCl pH 7.5 containing 1% FBS and 4 mM CaCl_2_). Digested PBMC were washed once in complete RPMI and then used in acute infection assays as described. TZM-bl cells (NIH AIDS Research and Reference Reagent Program, Germantown, MD) were maintained in DMEM (Invitrogen, Carlsbad, CA) containing 10% fetal bovine serum. CD8+ T cells were enriched via negative selection from PBMC with the EasySep enrichment kit (Stem Cell Technologies, Vancouver, Canada) and activated by either PHA (20 µg/mL, Sigma), PMA (0.05 µg/mL, Sigma) plus ionomycin (1 µg/mL, Sigma), or anti-CD3/CD28 antibody-loaded beads (T Cell Activation/Expansion Kit, Miltenyi Biotec, Auburn, CA), all in the presence of 50 U/mL of IL-2. After 3 days of activation, the cells were washed to remove stimuli, medium was replaced with complete RPMI supplemented with IL-2 at a cell density of 1×10^6^ cells/mL, and the culture supernatants were harvested at day 5 and 7 post-stimulation.

### ELISA for XCL1 production and GAG expression

CD8+ T-cell culture supernatants (after 3 days of activation and washing) were tested for XCL1 production using the Human XCL1/Lymphotactin DuoSet ELISA (R&D Systems). To confirm the efficiency of GAG removal following heparitinase digestion, a cell-based ELISA was performed according to a previously established protocol with some modifications [50]. Briefly, activated PBMC were washed in PBS and seeded at 5×10^4^ cells/well in 50 µL of PBS, and dried by evaporation at room temperature overnight. Plates were washed once with PBS and immediately fixed in ice-cold 2% paraformaldehyde at 4°C for 20 min, followed by washing in PBS. The wells were blocked in 0.2% casein-PBS buffer for 1 hr at 37°C, washed once with PBS, and incubated with 10 µg/mL of anti-heparan sulfate mAbs, clone 10E4 (AMSBIO, Lake Forest, CA) and clone T320.11 (EMD Millipore, Temecula, CA) for 2 hr at RT. An anti-CD4 mAb (RPA-T4, BD Biosciences) was used as a control for the non-specific effects of digestion on cell-surface protein expression (2 hr at RT). After 3 washes with PBS, the wells were incubated with polyclonal HRP-conjugated anti-mouse antibodies (Thermo Fisher Scientific, Rockford, IL). After 3 washes with PBS, wells were incubated with substrate solution until color development and immediate incubation with stop solution (R&D Systems), followed by reading optical density at 450 nm. Background measurements obtained with secondary antibody alone were subtracted from all readings.

### XCL1 and cell surface receptor interaction assays

The ability of XCL1 to downmodulate cell surface expression of CD4, CXCR4, and CCR5 was investigated by flow cytometry. Briefly, CD4+ T cells were cultured in the presence/absence of XCL1 at 20 µg/mL for 24 hours. Cells were then washed and stained for receptor expression using anti-CD4, CXCR4, and CCR5 mAbs (BD Biosciences, San Jose, CA). To further determine possible interactions between XCL1 and CD4, we assessed the ability of XCL1 to interfere with binding of different anti-CD4 mAbs targeted to different domains of CD4. Six fluorochrome-conjugated antibodies were used: OKT4 (eBioscience, San Diego, CA), 13B8 (Beckman Coulter, Inc., Indianapolis, IN), VIT4 (Miltenyi Biotec), RPA-T4, Leu3A/SK3, and L200 (all 3 from BD Biosciences). Two unlabeled mouse mAbs were used, DB-81 [51] and 9H5A8 (Novus Biologicals, Littleton, CO), followed by subsequent anti-mouse-R-phycoerythrin staining (Sigma). Enriched CD4+ T cells were incubated with XCL1 at 20 µg/mL or with PBS for 30 minutes at 4°C, without washing, cells were then stained with the various anti-CD4 mAbs listed. All data were acquired on a BD FACS Canto flow cytometer (San Jose, CA) and analyzed with FlowJo software version 9.5.2 for Macintosh (TreeStar, San Carlos, CA).

### HIV-1 isolates and infection assays

The HIV-1 isolates used in this study included two laboratory strains [IIIB (X4) and BaL (R5)], the dual-tropic primary isolate, 92HT599 (X4R5), and a set of primary isolates derived in our laboratory and minimally passaged *ex vivo* (98USSG, 07USLD, 07USPC, 08USSE, 97IT6366, 08USKD), obtained by culture of PBMC from chronically infected individuals. Acute cell-free HIV-1 infection was performed by addition of the viral stocks (50–100 pg of p24 Gag antigen per well) to duplicate cultures of activated PBMCs (PHA+IL-2 for 72 h) in round-bottom 96-well plates seeded at 2×10^5^ cells per well in RPMI+10% FBS+20 U/mL of IL-2, or to TZM-bl cells seeded in 24-well plates overnight at 5×10^4^ per well in DMEM+10% FBS for infection. Infected cells were cultured in the presence/absence of XCL1 at doses ranging from 0.06–1.5 µM. The levels of HIV-1 replication were assessed by measuring the extracellular release of p24 Gag protein in cell-free culture supernatants taken daily between days 3 and 7 post-infection using a highly sensitive Alpha (Amplified Luminescent Proximity Homogeneous Assay) technology immunoassay (AlphaLISA HIV p24 Research Immunoassay Kit, PerkinElmer, Waltham, MA). On day 7 of infection, cells were harvested for viability testing via absolute counting by flow cytometry. Cell viability was determined by normalization of the total live-gated cell counts in XCL1-treated wells to the number of cells recovered from control wells (untreated with XCL1). To show the physiological relevance of our infection data, we performed an infection assay whereby HIV-1 IIIB was pre-incubated (prior to addition to target cells) with XCL1 alone (1 µM) or with XCL1 combined with anti-XCL1 pAb (10 µg/mL of the same pAb used to block HIV-1 capture by XCL1). To control for non-specific effects of the pAb we also included control wells (no XCL1 treatment) with pAb alone.

As a test for specificity of the interaction between XCL1 and the HIV-1 envelope glycoprotein (gp120), we performed infection assays with GFP-expressing VSV-G pseudotyped virus provided by Michael P. Marino (CBER/FDA, Bethesda, MD, USA). PBMC (5×10^4^) were seeded in 96-well round bottom plates and infected in a 50 µL volume of pseudotyped virus (MOI of 10) in the presence or absence of XCL1 WT overnight, with each condition in quadruplicate wells. Wells were supplemented with an additional 50 µL of complete RPMI at 24 hr post-infection to yield a final well volume of 100 µL. Individual wells were harvested 48 hr post-infection for flow cytometry detection of GFP-positive (infected) cells. To supplement the data counting the absolute numbers of infected cells, mean fluorescence intensity was also determined to indicate the amount of virus infection within each GFP-positive event (infected cell).

### HIV-1 attachment and entry assay

The HIV-1 attachment and entry assays were performed on TZM-bl cells with the primary, dual-tropic HIV-1 isolate 92HT599. TZM-bl cells (10^6^ per replicate; two replicates per treatment) were seeded in 12-well plates overnight to achieve a confluent cell monolayer. Without disturbing the monolayer, cells were washed with PBS to remove media, followed by pre-incubation for 15 min at room temperature with XCL1 diluted in PBS, and then exposed to 500 µL of the undiluted viral stock (124 ng/mL of p24) for 4 h (attachment) or 6 h (entry) at 37°C, in the continuous presence of XCL1. Two wells of untreated cells were incubated for 4 hr with virus at 4°C to determine the background signal level (trypsin-insensitive despite low-temperature conditions preventing virus entry). As specificity controls, replicate wells were pretreated with known inhibitors/inducers of viral attachment/entry prior to virus incubation: CXCL4/PF4 at 15 µg/mL (R&D Systems), peptide T-20 at 50 µg/mL (NIH AIDS Research and Reference Reagent Program, Germantown, MD), an anti-CD4 mAb at 20 µg/mL (azide-free RPA-T4, eBioscience, San Diego, CA), or CCL5/RANTES at 15 µg/mL (R&D Systems). After incubation, the cells were washed with PBS to remove unbound virus, without disturbing the cell monolayer. Entry assay wells were treated with pre-warmed bovine trypsin (Sigma) for 5 min at 37°C, followed by trypsin inactivation with cold DMEM medium containing 10% (vol/vol) FBS. Both trypsin-treated (entry assay) and untreated (attachment assay) cells were then washed two times with cold PBS, and lysed with 100 µL of 0.5% (wt/vol) Triton X-100 to quantify the amount of cell-associated p24 protein. The specific signal was calculated by subtracting the background p24 levels measured in wells incubated at 4°C (treated with trypsin) from the p24 levels measured in each test sample.

### HIV-1 virion capture assay

The virion capture assay was performed as previously described [14]. Briefly, immunomagnetic beads (4×10^4^ per tube) covalently linked to a polyclonal antiserum to rabbit IgG (Invitrogen) were incubated with a polyclonal rabbit IgG antibody to human XCL1 (Peprotech), washed with PBS containing 0.05% (wt/vol) bovine casein and then loaded with recombinant human XCL1 (2.5 µg per reaction). After removing unbound XCL1 by repeated PBS washes, chemokine-armed beads were incubated with 0.5 mL of the viral stock (HIV-1 IIIB (X4); 20 ng of p24 Gag protein/test). To test the specificity of XCL1 interaction with the virus, the XCL1-armed immunomagnetic beads were pre-incubated with monoclonal (mAb) and polyclonal (pAb) anti-XCL1 antibodies (R&D Systems, 20 µg/mL) for 10 minutes at room temperature prior to virus addition. After incubation with virus for 1 h at room temperature, the beads were washed to remove unbound virus particles and treated with 0.5% Triton X-100 to lyse the captured virions. The amount of captured p24 Gag protein was quantified by AlphaLISA®.

As an additional measure for the exclusive interaction between XCL1 and the HIV-1 envelope glycoprotein (gp120), we assessed the ability of XCL1 to capture VSV-G pseudotyped virus. Anti-mouse immunomagnetic beads were armed with monoclonal anti-VSV-G antibody (KeraFAST Inc., Boston, MA) to show the efficiency of VSV-G pseudotyped virus capture in our experimental design. In parallel, anti-rabbit immunomagnetic beads armed with both anti-XCL1 pAb and subsequent XCL1 WT were tested for the ability to capture VSV-G-pseudotyped virus. For accurate comparison of capture between beads armed with anti-VSV-G mAb and beads armed with XCL1, equal amounts of VSV-G-pseudotyped virus was added to all capture reactions.

### Co-immunoprecipitation

To evaluate the direct interaction between XCL1 and the gp120 external envelope glycoprotein, we performed co-immunoprecipitation experiments using purified YU2 gp120 protein. To assess this interaction, XCL1 WT, W55D and CC3 proteins were biotinylated using the LYNX Rapid Conjugation Kit (AbD Serotec, Kidlington, UK). In two conditions we assessed the specificity of XCL1-gp120 interactions via pre-incubation of biotinylated XCL1 WT with 5 µg of anti-XCL1 pAb (R&D Sytems) or goat IgG (R&D Systems), as a control, for 1 h in 100 µL of PBS. Following the presence or absence of antibody pre-incubation, a mixture of biotinylated XCL1 WT, W55D, or CC3 (2 µg) was incubated with gp120 (2 µg) in 100 µL of PBS+0.2% casein for 3 h at room temperature with constant rotation. Subsequent incubation with 50 µL of streptavidin-coated magnetic beads (Invitrogen) in 200 µL of RIPA buffer was performed for an additional 10 min incubation. The samples were washed three times with RIPA buffer, dissolved in SDS loading buffer, and loaded on 12% polyacrylamide gels and resolved by SDS gel electrophoresis. Protein was transferred onto nitrocellulose membranes and blotted with an anti-gp120 mAb (b24; a gift from George K. Lewis, University of Maryland, Baltimore, MD).

### Ethics statement

Anonymized samples of peripheral blood were obtained from healthy volunteer donors at the NIH Blood Bank under a protocol approved by the NIH IRB.

## Supporting Information

Figure S1 **Conformation-dependent XCL1 inhibition is irrespective of coreceptor specificity and target cell type.** To demonstrate the broad range and reproducibility of XCL1 conformation-dependent inhibition, we showed the dependency on the all-β/alternatively-folded XCL1 structure to inhibit X4-strains (IIIB) and R5-strains (BaL) in TZM-bl cells. We also confirmed that the locked, chemokine-folded XCL1 variant (CC3) exhibited minimal inhibitory effect.
(TIFF)

Figure S2 **HIV-1 inhibition by XCL1 is reversed in the presence of anti-XCL1 neutralizing antibody.** PBMC infected with HIV-1 IIIB were cultured with medium alone as a control (grey bar) or in the presence of 1 µM of XCL1 WT (green bar). In parallel, XCL1 WT was pre-incubated with goat polyclonal anti-XCL1, followed by incubation with the virus and subsequent addition to target cells (XCL1+pAb). As control, virus was pre-incubated with pAb alone and then added to target cells (pAb alone). Virus replication was assessed by p24 AlphaLISA immunoassay. Data shown are a percentage of the HIV-1 p24 produced by untreated cells (Control, grey bar).
(TIFF)

Figure S3 **XCL1 does not inhibit infection with VSV-G pseudotyped virus.** Infection of PBMC with GFP-expressing VSV-G-pseudotyped virus was unaffected by a dose-response treatment with XCL1 WT. The amount of virus infection was quantified by the mean fluorescence intensity of the gated infected cells from the total PBMC harvested from each well.
(TIFF)

Figure S4 **Digestion with heparitinase reduces cell-surface heparan sulfate expression without affecting CD4 expression.** PBMC were incubated in the presence (‘Heparitinase-treated’) or absence (‘Untreated’) of heparitinase to digest cell surface GAG expression, followed by adsorption of the cells to flat-bottom microtiter plates. Subsequent incubation with mAbs was performed in a cell-based ELISA protocol. As a control for the non-specific effect of heparitinase on cell-surface protein expression, wells were incubated with an anti-CD4 mAb (RPA-T4) (left panel). To evaluate the efficacy of heparan sulfate removal, we used two different anti-heparan sulfate mAbs, 10E4 (center panel) and T320.11 (right panel). Data represent the mean (±SD) OD readings at 450 nm from triplicate wells after subtraction of background readings obtained with secondary antibody alone.
(TIFF)
