# Supplementary figures and images for: The CD8-Derived Chemokine XCL1/Lymphotactin Is a Conformation-Dependent, Broad-Spectrum Inhibitor of HIV-1
Source: PLoS Pathog. 2013 Dec 26;9(12):e1003852. doi: 10.1371/journal.ppat.1003852 (PMC3873461; doi:10.1371/journal.ppat.1003852)

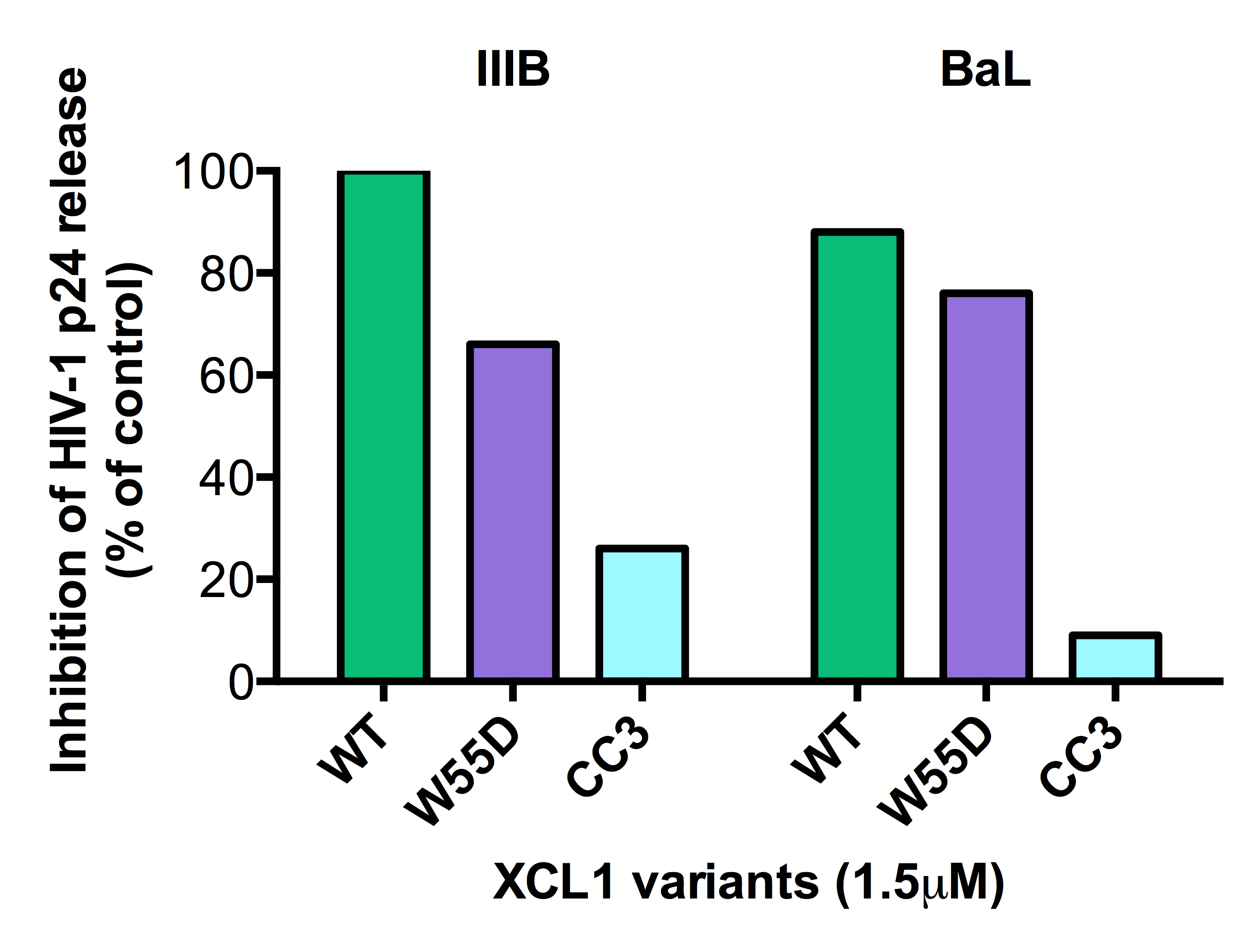

Supplement: Figure S1 — Conformation-dependent XCL1 inhibition is irrespective of coreceptor specificity and target cell type. To demonstrate the broad range and reproducibility of XCL1 conformation-dependent inhibition, we showed the dependency on the all-β/alternatively-folded XCL1 structure to inhibit X4-strains (IIIB) and R5-strains (BaL) in TZM-bl cells. We also confirmed that the locked, chemokine-folded XCL1 variant (CC3) exhibited minimal inhibitory effect. (TIFF) [file ppat.1003852.s001.tiff]

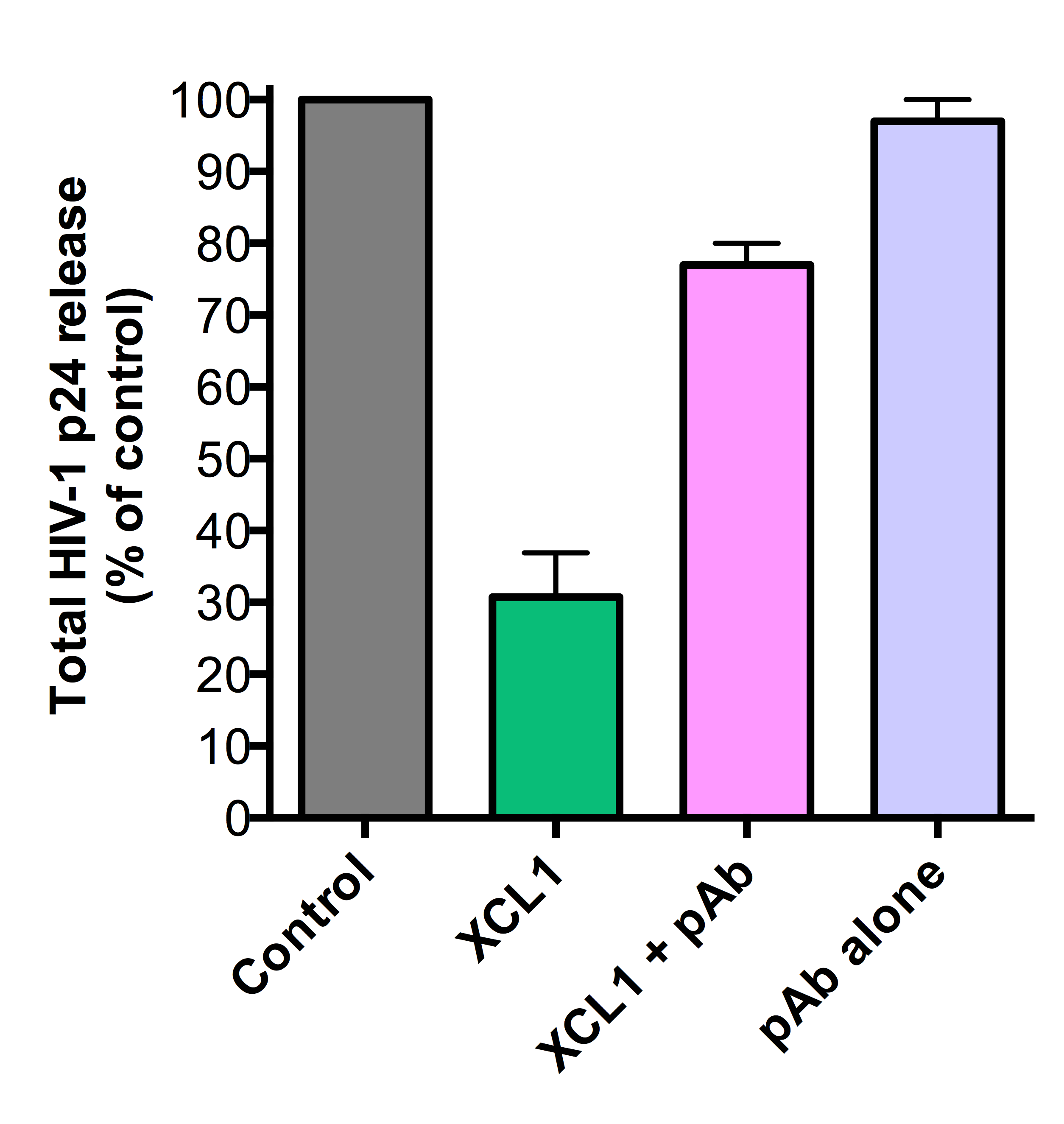

Supplement: Figure S2 — HIV-1 inhibition by XCL1 is reversed in the presence of anti-XCL1 neutralizing antibody. PBMC infected with HIV-1 IIIB were cultured with medium alone as a control (grey bar) or in the presence of 1 µM of XCL1 WT (green bar). In parallel, XCL1 WT was pre-incubated with goat polyclonal anti-XCL1, followed by incubation with the virus and subsequent addition to target cells (XCL1+pAb). As control, virus was pre-incubated with pAb alone and then added to target cells (pAb alone). Virus replication was assessed by p24 AlphaLISA immunoassay. Data shown are a percentage of the HIV-1 p24 produced by untreated cells (Control, grey bar). (TIFF) [file ppat.1003852.s002.tiff]

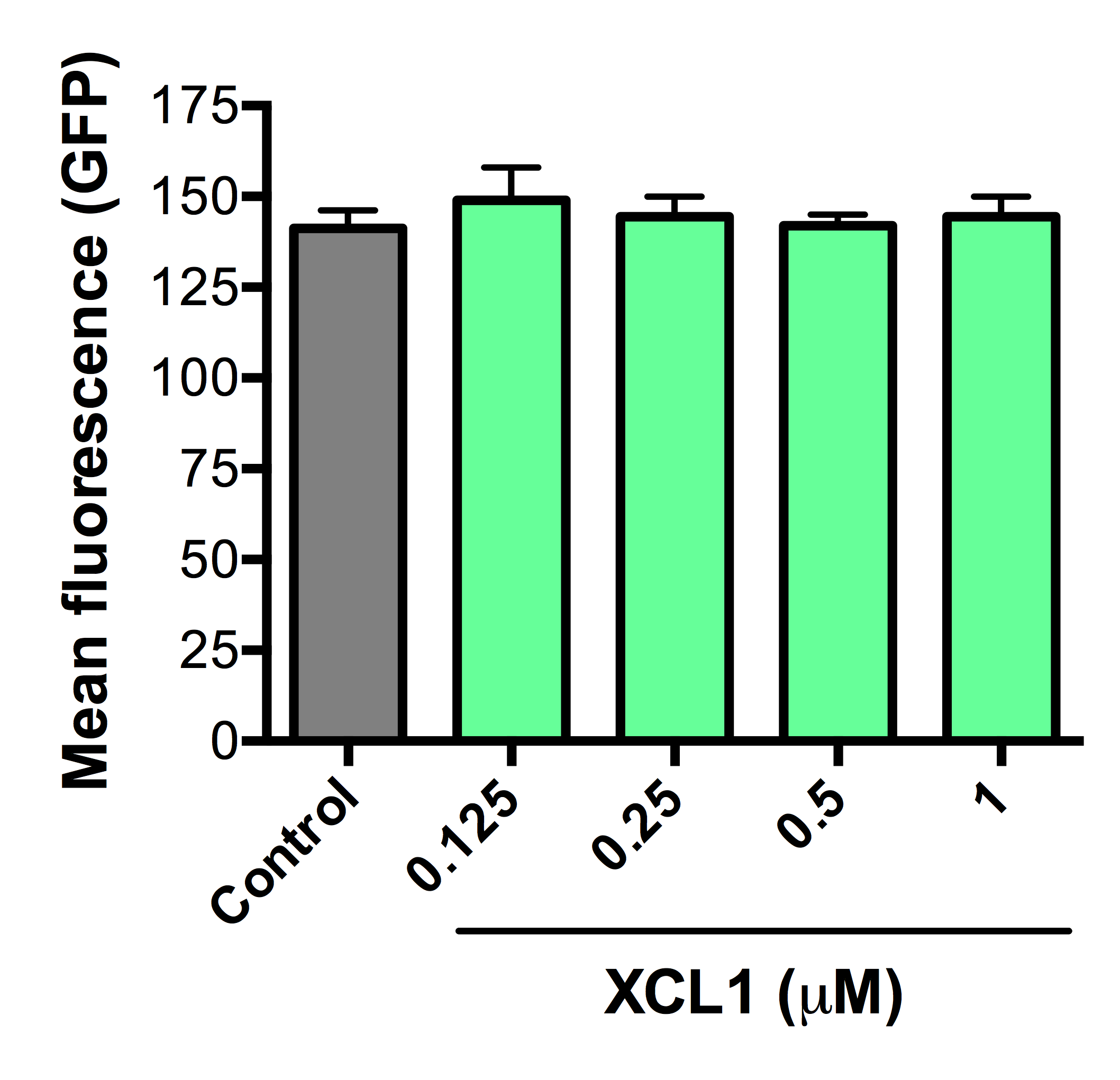

Supplement: Figure S3 — XCL1 does not inhibit infection with VSV-G pseudotyped virus. Infection of PBMC with GFP-expressing VSV-G-pseudotyped virus was unaffected by a dose-response treatment with XCL1 WT. The amount of virus infection was quantified by the mean fluorescence intensity of the gated infected cells from the total PBMC harvested from each well. (TIFF) [file ppat.1003852.s003.tiff]

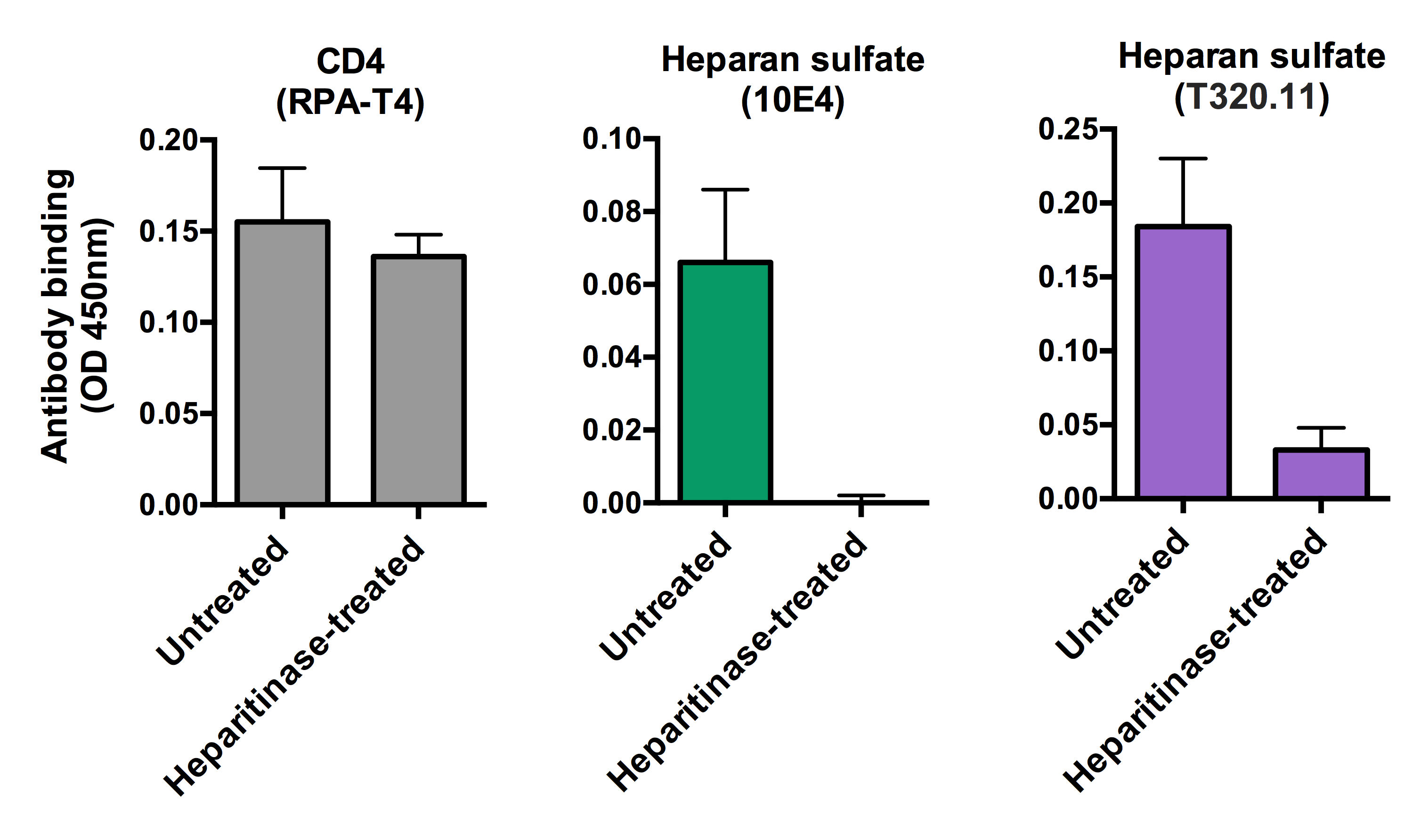

Supplement: Figure S4 — Digestion with heparitinase reduces cell-surface heparan sulfate expression without affecting CD4 expression. PBMC were incubated in the presence (‘Heparitinase-treated’) or absence (‘Untreated’) of heparitinase to digest cell surface GAG expression, followed by adsorption of the cells to flat-bottom microtiter plates. Subsequent incubation with mAbs was performed in a cell-based ELISA protocol. As a control for the non-specific effect of heparitinase on cell-surface protein expression, wells were incubated with an anti-CD4 mAb (RPA-T4) (left panel). To evaluate the efficacy of heparan sulfate removal, we used two different anti-heparan sulfate mAbs, 10E4 (center panel) and T320.11 (right panel). Data represent the mean (±SD) OD readings at 450 nm from triplicate wells after subtraction of background readings obtained with secondary antibody alone. (TIFF) [file ppat.1003852.s004.tiff]
